# Virtual Visits for Acute, Nonurgent Care: A Claims Analysis of Episode-Level Utilization

**DOI:** 10.2196/jmir.6783

**Published:** 2017-02-17

**Authors:** Aliza S Gordon, Wallace C Adamson, Andrea R DeVries

**Affiliations:** ^1^ HealthCore, Inc Wilmington, DE United States; ^2^ Anthem, Inc Indianapolis, IN United States

**Keywords:** virtual visit, health care utilization, claims analysis

## Abstract

**Background:**

Expansion of virtual health care—real-time video consultation with a physician via the Internet—will continue as use of mobile devices and patient demand for immediate, convenient access to care grow.

**Objective:**

The objective of the study is to analyze the care provided and the cost of virtual visits over a 3-week episode compared with in-person visits to retail health clinics (RHC), urgent care centers (UCC), emergency departments (ED), or primary care physicians (PCP) for acute, nonurgent conditions.

**Methods:**

A cross-sectional, retrospective analysis of claims from a large commercial health insurer was performed to compare care and cost of patients receiving care via virtual visits for a condition of interest (sinusitis, upper respiratory infection, urinary tract infection, conjunctivitis, bronchitis, pharyngitis, influenza, cough, dermatitis, digestive symptom, or ear pain) matched to those receiving care for similar conditions in other settings. An episode was defined as the index visit plus 3 weeks following. Patients were children and adults younger than 65 years of age without serious chronic conditions. Visits were classified according to the setting where the visit occurred. Care provided was assessed by follow-up outpatient visits, ED visits, or hospitalizations; laboratory tests or imaging performed; and antibiotic use after the initial visit. Episode costs included the cost of the initial visit, subsequent medical care, and pharmacy.

**Results:**

A total of 59,945 visits were included in the analysis (4635 virtual visits and 55,310 nonvirtual visits). Virtual visit episodes had similar follow-up outpatient visit rates (28.09%) as PCP (28.10%, *P*=.99) and RHC visits (28.59%, *P*=.51). During the episode, lab rates for virtual visits (12.56%) were lower than in-person locations (RHC: 36.79%, *P*<.001; UCC: 39.01%, *P*<.001; ED: 53.15%, *P*<.001; PCP: 37.40%, *P*<.001), and imaging rates for virtual visits (6.62%) were typically lower than in-person locations (RHC: 5.97%, *P*=.11; UCC: 8.77%, *P*<.001; ED: 43.06%, *P*<.001; PCP: 11.26%, *P*<.001). RHC, UCC, ED, and PCP were estimated to be $36, $153, $1735, and $162 more expensive than virtual visit episodes, respectively, including medical and pharmacy costs.

**Conclusions:**

Virtual care appears to be a low-cost alternative to care administered in other settings with lower testing rates. The similar follow-up rate suggests adequate clinical resolution and that patients are not using virtual visits as a first step before seeking in-person care.

## Introduction

Health care delivery is moving outside traditional settings of physician offices and emergency departments (EDs) into convenient quick-care sites [1] such as retail health clinics (RHCs) and urgent care centers (UCCs). In addition to these relatively established alternatives for nonurgent acute care [2,3], venues maximizing the latest technology are emerging. Telehealth includes both asynchronous structured e-visits and online synchronous live video visits (virtual visits). Structured e-visits refer to online communication (eg, inputting symptoms into a website) and provision of treatment plans over the Internet, while virtual visits feature real-time video consultation. By providing a means for patients to receive health care from any location at all hours, telehealth platforms expand patient access to medical care [1,4,5]. While the American College of Physicians supports telemedicine, its policy statement stresses the importance of maintaining the physician-patient relationship [6].

Because telehealth and particularly virtual visits are relatively new care options, published literature is lacking, with previous studies largely focused on acceptability or patient characteristics rather than outcomes [7-11]. Quality issues are particularly concerning for telehealth, such as whether physicians can provide accurate diagnoses without hands-on physical examinations of patients, whether patients receive appropriate laboratory testing after the visit, and whether antibiotics are overprescribed [12-14]. There are also concerns about whether the visits are truly efficient—if additional follow-up visits occur as a result of unresolved symptoms or because patients used online visits as a first opinion before seeking care in an office setting, this would diminish the apparent cost-saving potential. The few studies that examined the quality of virtual visits found higher antibiotic use after virtual visits compared with in-person office visits [13]—including prescription of broad-spectrum antibiotics [14]—for acute respiratory infections where antibiotics are not generally recommended. Providers of virtual visits were less likely than those in offices to order diagnostic tests to determine whether cases of pharyngitis were bacterial or viral, which is considered the standard of care [13]. Using follow-up visits as a proxy measure of misdiagnoses or treatment failures, researchers found a similar rate among patients participating in structured e-visits as those going to an office [6,12]. While an initial telehealth visit (e-visit or virtual) is usually less costly than an in-person visit, few if any studies analyzed episode-level costs associated with virtual visits (including follow-up care), although 1 study of structured e-visits found treatment costs per episode of care to be lower than traditional settings [7].

Virtual visits have recently become available through independent online health care delivery sites that are often covered by patients’ health plans [14]. Starting in 2014, virtual visits became available to the members in this study as a covered benefit, with patient copays similar to primary care physicians (PCP) office visits. After patients create an initial profile, they choose from a list of available providers licensed in their state of residence [15]. Physicians are reimbursed $49 per virtual visit. The service has become increasingly popular with members, who received up to 4000 virtual visits per month through this service in 2015. Patient satisfaction has been high, with a net promoter score of 65%, based on an exit survey of patients who used the service administered as part of the health plan’s virtual care program (personal communication, W Adamson).

To expand our understanding of the care provided and costs associated with virtual health care, we examined the care for specific acute, nonurgent conditions (eg, colds, allergies, urinary tract infections) provided by physicians via a virtual visit platform. Care provided through virtual visits, including subsequent care during a 3-week follow-up period, was compared with care delivered in the RHC, UCC, ED, and PCP office settings. This study is unique in assessing care and costs of virtual visit episodes, in contrast to previous studies of telehealth costs that have assessed structured e-visits only or have not taken into account follow-up care after the initial visit.

## Methods

### Study Design

This cross-sectional retrospective study used data from commercially insured members receiving virtual care matched to members receiving care for similar conditions in other settings. The claims-based dataset was derived from the HealthCore Integrated Research Database (HIRD), a large administrative claims database containing medical and pharmacy claims for 14 Anthem commercial health plans geographically dispersed across the United States. The patient sample was identified from claims with service dates during the study period, January 1, 2014, through May 11, 2015. Researchers had access to a limited dataset containing no patient identifiers. This study was conducted in full compliance with the Health Insurance Portability and Accountability Act. This study was nonexperimental and was exempt from investigational review board approval.

The index date was defined as the date of the first outpatient or ED claim in a 3-week period for 11 of the most commonly diagnosed conditions through the telehealth platform: sinusitis, upper respiratory infections (URIs), urinary tract infections (UTIs), conjunctivitis, bronchitis, pharyngitis, influenza, cough, dermatitis, nausea/vomiting/diarrhea, and ear pain, based on *International Classification of Diseases, Ninth Revision* (ICD-9) diagnosis codes (Multimedia Appendix 1). Baseline patient characteristics were determined from claims during the 6 months prior to the index date.

### Selection Criteria

The study included adults younger than 65 years of age and children who had health plan eligibility for at least 6 months before and 3 weeks after the index date. Patients with serious or expensive health conditions, defined by Deyo-Charlson Comorbidity Index (DCI) scores of greater than 2, or with cystic fibrosis, transplant, end-stage renal disease, HIV, hemophilia, stroke, or respiratory failure were excluded.

### Episode Identification

Visits for conditions of interest were classified according to the setting where the visit occurred: virtual (identified by Current Procedural Terminology [CPT] code 99444 and tax ID, representing all covered telehealth visits), RHCs (identified by tax ID and National Provider Identifier [NPI] numbers), UCCs (identified by tax ID and NPI numbers; only large national UCC chains included), EDs (identified by revenue codes and CPT codes), and PCP offices (identified by CPT codes for outpatient evaluation and management visits with provider specialty noted as primary care, internal medicine, general medicine, or pediatrics).

An episode was defined as the index visit plus 3 weeks following. If patients had 2 or more potential index visits less than 3 weeks apart, only the first visit was used to identify an episode. If patients had more than 1 visit on the same day, a hierarchy was used to determine the index visit (as opposed to follow-up visit). The hierarchy was virtual > RHC > PCP > UCC > ED; this order was chosen due to likelihood of patients going to a more “urgent” care location after a different option was tried if more than 1 location was visited in a single day.

### Patient Selection

Members receiving care from RHC, UCC, ED, and PCP offices were matched to those with virtual visits in a 3:1 ratio for each location (to increase statistical power) on acute condition, quarter and year of index date, state/region of residence, and child (<18 years) or adult age group.

### Care and Cost Outcome Measures

The primary outcome measures were care provided (utilization during and following the visit) and cost of care. The follow-up period for outcomes assessment was from the index date to 3 weeks after to allow sufficient time for most minor conditions to resolve [16]. The follow-up period for antibiotic fills was 3 days from the index date since most antibiotic fills occurred during this time and fills occurring later in the episode may have occurred after a follow-up visit at a different location.

Care provided was assessed by subsequent medical care after the initial visit (ie, outpatient evaluation and management visit [follow-up visit], ED visit, or inpatient hospitalization), laboratory tests performed, imaging performed, and antibiotic fill rates and use of broad-spectrum antibiotics, for patients where pharmacy data were available. Allowed cost per episode included the cost of the initial visit, subsequent medical care, and pharmacy costs.

All care and costs during the 3-week episode were included, not just those for care with the same diagnosis as the index visit, since it is difficult to determine whether subsequent care is related to the initial visit (eg, pneumonia can develop after a different infection).

### Statistical Analysis

The outcome measures were analyzed to determine differences between virtual visits and other locations of care. A significance level of α<.05 (2-sided) comparing each location with virtual was considered for all analyses (*P* values compare each location to virtual). Frequencies were reported and χ^2^ tests were used for all differences in care patterns/utilization. General linear models with gamma distribution and log link were used to compare costs and were adjusted to account for differences in age category and common baseline comorbidities (see Table 2 for specific age categories and comorbidities). We did not adjust for prior costs or utilization since prior care seeking is related to future care seeking behavior and does not necessarily match with health status. Due to the large variation in and skewed nature of follow-up costs, a sensitivity analysis was performed using winsorized values at the 5% and 95% level. Statistical analyses were conducted using SAS version 9.4 (SAS Institute, Inc).

## Results

### Selected Visits

**Figure 1:**
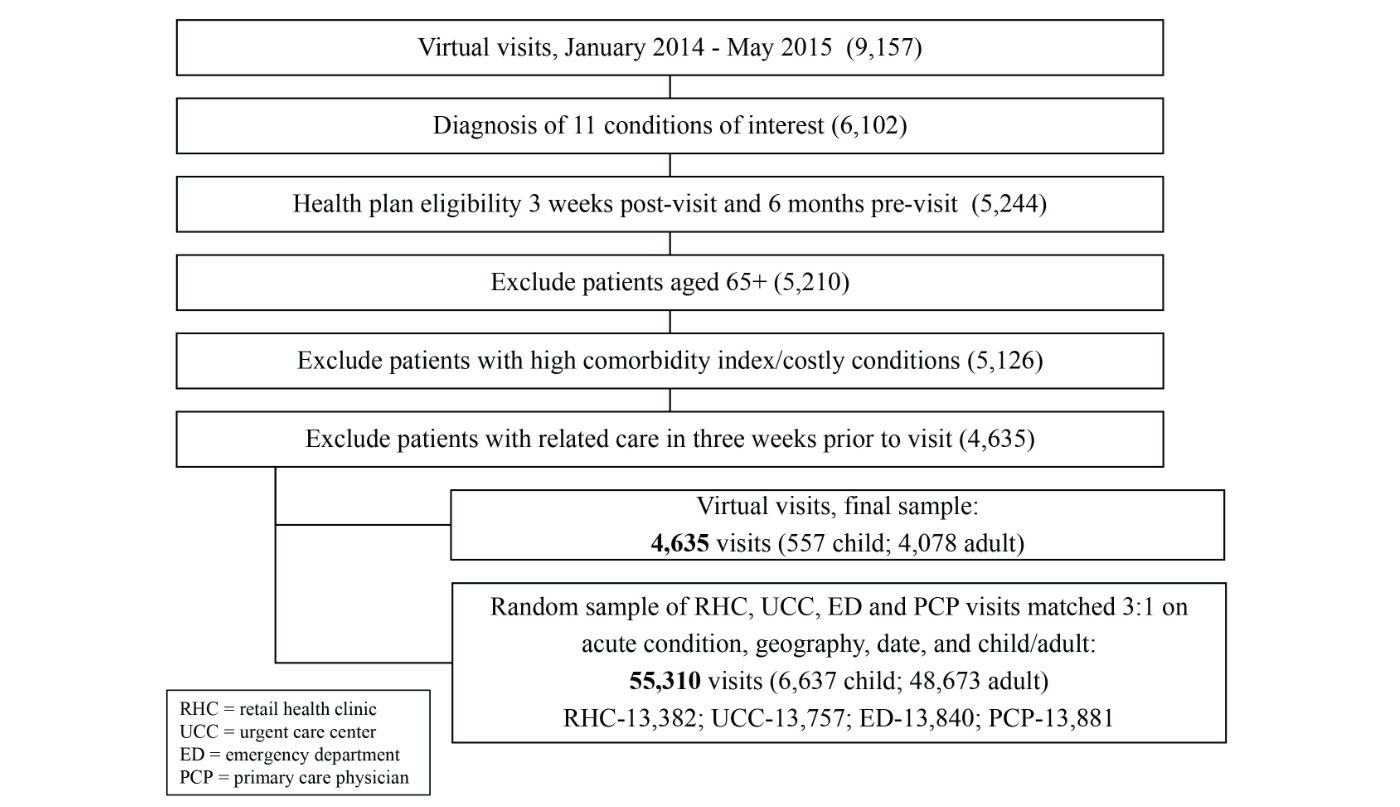
Attrition; number of virtual visits at each step.

A total of 4635 virtual and 55,310 in-person visits were included in the analysis (13,832 RHC; 13,757 UCC; 13,840 ED; 13,881 PCP; see Figure 1 and Table 1). Pharmacy data were available for 3182 virtual and 29,562 other visits (7518 RHC; 7188 UCC; 7227 ED; 7629 PCP). The condition mix in each group consisted of the proportion of virtual visits with each of the 11 conditions (Table 1). Sinusitis and URI were the most highly represented conditions, accounting for more than half of the sample.

**Table 1:** Visits by diagnosis.

Diagnosis	Virtual, n (%)	RHC^a^, n (%)	UCC^b^, n (%)	ED^c^, n (%)	PCP^d^, n (%)
Sinusitis	1689 (36.44)	5055 (36.55)	5062 (36.80)	5029 (36.34)	5060 (36.45)
Upper respiratory infection	849 (18.32)	2540 (18.36)	2534 (18.42)	2537 (18.33)	2541 (18.31)
Urinary tract infection	413 (8.91)	1240 (8.96)	1238 (9.00)	1236 (8.93)	1239 (8.93)
Bronchitis	397 (8.57)	1191 (8.61)	1195 (8.69)	1188 (8.58)	1192 (8.59)
Conjunctivitis	356 (7.68)	1070 (7.74)	1035 (7.52)	1071 (7.74)	1068 (7.69)
Pharyngitis	285 (6.15)	854 (6.17)	853 (6.20)	851 (6.15)	853 (6.15)
Cough	158 (3.41)	471 (3.42)	473 (3.44)	469 (3.39)	472 (3.40)
Contact dermatitis	145 (3.13)	435 (3.14)	410 (2.98)	432 (3.12)	432 (3.11)
Influenza	140 (3.02)	418 (3.02)	386 (2.81)	419 (3.03)	417 (3.00)
Digestive symptoms—diarrhea, nausea, vomiting	104 (2.24)	260 (1.88)	310 (2.25)	312 (2.25)	311 (2.24)
Ear disorders—ear pain	99 (2.14)	296 (2.14)	261 (1.90)	296 (2.14)	296 (2.13)
Total	4635 (100)	13,832 (100)	13,757 (100)	13,840 (100)	13,881 (100)

^a^RHC: retail health clinic.

^b^UCC: urgent care center.

^c^ED: emergency department.

^d^PCP: primary care physician.

### Baseline Patient Characteristics

In the RHC, UCC, and ED groups, the highest proportion of patients were 18 to 34 years of age, whereas the highest proportion of virtual patients were 35 to 49 years, and 50 to 64 years in the PCP group (Table 2). The majority of patients in all groups were women and had a low disease burden, indicated by a DCI score of zero. Of those with comorbidities, the most common was hypertension. While still low, DCI score and common comorbidities were highest among the PCP group (12% of the PCP group had DCI score of 1 or 2, compared with 10% of virtual and 7% of other groups).

**Table 2:** Baseline characteristics.

		Virtual, 4635	RHC^a^, 13,832		UCC^b^, 13,757		ED^c^, 13,840		PCP^d^, 13,881	
		mean (SD) / n (%)	mean (SD) / n (%)	*P* ^e^	mean (SD) / n (%)	*P* ^e^	mean (SD) / n (%)	*P* ^e^	mean (SD) / n (%)	*P* ^e^
Age of adults, mean (SD)		40.1 (10.8)	39.3 (12.7)	<.001	37.5 (13.1)	<.001	38.1 (13.5)	<.001	42.7 (13.2)	<.001
Age of children, mean (SD)		8.4 (5.2)	9.8 (4.7)	<.001	9.4 (5.1)	<.001	7.1 (5.4)	<.001	7.1 (5.1)	<.001
**Age category, n (%)**				<.001		<.001		<.001		<.001
	<18	557 (12.0)	1664 (12.0)		1622 (11.8)		1676 (12.1)		1675 (12.1)	
	18-34	1414 (30.5)	4814 (34.8)		5510 (40.1)		5355 (38.7)		3543 (25.5)	
	35-49	1729 (37.3)	4248 (30.7)		3912 (28.4)		3784 (27.3)		4188 (30.2)	
	50-64	935 (20.2)	3106 (22.5)		2713 (19.7)		3025 (21.9)		4475 (32.2)	
Female, n (%)		2837 (61.2)	9143 (66.1)	<.001	5221 (62.1)	.31	8111 (58.6)	.002	8472 (61.0)	.83
**Deyo-Charlson Index Score, n (%)**				<.001		<.001		<.001		<.001
	0	4174 (90.1)	12,856 (92.9)		12,802 (93.1)		12,828 (92.7)		12,205 (87.9)	
	1	273 (5.9)	540 (3.9)		476 (3.5)		354 (2.6)		911 (6.6)	
	2	188 (4.1)	436 (3.2)		479 (3.5)		658 (4.8)		765 (5.5)	
**Comorbidities, n (%)**										
	Diabetes mellitus	127 (2.7)	308 (2.2)	.05	288 (2.1)	.01	329 (2.4)	.17	560 (4.0)	<.001
	Hypertension	390 (8.4)	1081 (7.8)	.19	1147 (8.3)	.87	1620 (11.7)	<.001	1986 (14.3)	<.001
	Ischemic heart disease	23 (0.5)	91 (0.7)	.19	93 (0.7)	.18	142 (1.0)	.001	184 (1.3)	<.001
	Congestive heart failure	4 (0.1)	7 (0.1)	.39	12 (0.1)	.99	20 (0.1)	.34	14 (0.1)	.78
	Chronic obstructive pulmonary disease	23 (0.5)	30 (0.2)	.002	32 (0.2)	.005	66 (0.5)	.87	127 (0.9)	.01
	Asthma	190 (4.1)	380 (2.7)	<.001	409 (3.0)	<.001	443 (3.2)	.004	596 (4.3)	.70

^a^RHC: retail health clinic.

^b^UCC: urgent care center.

^c^ED: emergency department.

^d^PCP: primary care physician.

^e^*P* values show level of significance of the differences between each location versus virtual visits.

### Care Comparisons

Subsequent outpatient medical care after the initial visit was similar between virtual visits and other treatment settings. The percentage of follow-up visits within 3 weeks of the index visit, which is a potential indicator of misdiagnosis or treatment failure, was similar between the virtual (28.09%), RHC (28.59%; *P*=.54), and PCP groups (28.10%; *P*=.96; Table 3 **)**. While the UCC group had slightly fewer follow-up visits (25.62%; *P*<.001), the ED group had more (34.19%; *P*<.001). The virtual group had fewer ED visits within 3 weeks of the index visit (1.32%) compared with the UCC (2.68%; *P*<.001), ED (6.47%; *P*<.001), and PCP groups (1.84%; *P*=.02) but similar to the RHC group (1.61%; *P*=.14). The percentage of hospitalizations within 3 weeks followed a similar pattern, with the percentage for the virtual group (0.15%) lower than the UCC (0.41%; *P*=.01), ED (0.96%; *P*<.001), and PCP groups (0.37%; *P*=.02) and similar to the RHC group (0.28%; *P*=.12).

**Table 3:** Care patterns.

		Virtual	RHC^a^		UCC^b^		ED^c^		PCP^d^	
		n (%)	n (%)	*P* ^e^	n (%)	*P* ^e^	n (%)	*P* ^e^	n (%)	*P* ^e^
**All-cause follow-up care within 21 days of index visit, all conditions**										
	Outpatient evaluation and management visit	1302 (28.1)	3955 (28.6)	.51	3525 (25.6)	.001	4732 (34.2)	<.001	3900 (28.1)	.99
	ED visit	61 (1.3)	223 (1.6)	.16	368 (2.7)	<.001	895 (6.5)	<.001	255 (1.8)	.02
	Inpatient visit	7 (0.2)	39 (0.3)	.12	57 (0.4)	.01	133 (1.0)	<.001	52 (0.4)	.02
**Lab tests within 21 days, all conditions**		582 (12.6)	5089 (36.8)	<.001	5367 (39.0)	<.001	7356 (53.2)	<.001	5192 (37.4)	<.001
	UTI	85 (20.6)	1085 (87.5)	<.001	1189 (96.0)	<.001	1222 (98.9)	<.001	1095 (88.4)	<.001
	Pharyngitis	45 (15.8)	770 (90.2)	<.001	719 (84.3)	<.001	560 (65.8)	<.001	627 (73.5)	<.001
	Sinusitis	185 (11.0)	949 (18.8)	<.001	1243 (24.6)	<.001	2351 (46.8)	<.001	1302 (25.7)	<.001
	Bronchitis	40 (10.1)	285 (23.9)	<.001	271 (22.7)	<.001	648 (54.6)	<.001	308 (25.8)	<.001
**Imaging rates within 21 days, all conditions**		307 (6.6)	826 (6.0)	.11	1207 (8.8)	<.001	5960 (43.1)	<.001	1563 (11.3)	<.001
	Cough	18 (11.4)	46 (9.7)	.55	106 (22.4)	.003	397 (84.6)	<.001	111 (23.5)	.001
	Bronchitis	34 (8.6)	114 (9.6)	.59	193 (16.2)	<.001	844 (71.0)	<.001	212 (17.8)	<.001
	UTI	34 (8.2)	85 (6.9)	.35	132 (10.7)	.16	763 (61.7)	<.001	227 (18.3)	<.001
	URI	69 (8.1)	144 (5.7)	.01	203 (8.0)	.91	1067 (42.1)	<.001	236 (9.3)	.31
	Sinusitis	90 (5.3)	287 (5.7)	.59	358 (7.1)	.01	2152 (42.8)	<.001	497 (9.8)	<.001
**Antibiotic fill rates within 3 days^f^**										
	Any of the 6 infections below	1918 (70.5)	4193 (64.2)	<.001	4243 (67.9)	.02	3534 (56.7)	<.001	4477 (68.2)	.03
	Sinusitis	971 (83.9)	2340 (86.3)	.06	2084 (79.2)	.001	1835 (67.8)	<.001	2327 (82.9)	.42
	Pharyngitis	130 (74.3)	138 (29.6)	<.001	236 (53.8)	<.001	199 (46.4)	<.001	249 (53.7)	<.001
	Bronchitis	191 (68.5)	278 (40.8)	<.001	521 (76.4)	.01	393 (62.1)	.06	545 (78.1)	.002
	Conjunctivitis	157 (63.8)	463 (78.6)	<.001	363 (64.9)	.76	278 (51.8)	.002	373 (61.1)	.47
	UTI	217 (76.4)	628 (90.5)	<.001	473 (74.0)	.44	419 (65.6)	.001	415 (62.6)	<.001
	URI	252 (43.5)	346 (24.9)	<.001	566 (43.7)	.30	410 (31.9)	<.001	568 (43.0)	.82
**Antibiotic type**										
	Broad-spectrum antibiotic as first-line treatment^g^	1219 (69.0)	2299 (60.0)	<.001	2561 (66.3)	.04	1961 (61.9)	<.001	2704 (69.3)	.82

^a^ED: emergency department.

^b^PCP: primary care physician.

^c^RHC: retail health clinic.

^d^UCC: urgent care center.

^e^*P* values show level of significance of the differences between each location versus virtual visits.

^f^Sample includes patients with the condition of interest and pharmacy coverage.

^g^Sample includes patients with antibiotics fill without history of antibiotic use in prior 60 days.

Overall laboratory tests within 3 weeks of the index date (including during the initial visit for nonvirtual visits) were lower for the virtual group (12.56%) compared with RHC (36.79%; *P*<.001), UCC (39.01 %; *P*<.001), ED (53.15%; *P*<.001), and PCP (37.40%; *P*<.001; Table 3). Lab testing was particularly low in virtual episodes compared with other locations of care for pharyngitis and UTI, where testing for a bacterial infection is common. Overall imaging rates were similar between the virtual and RHC groups (6.62% vs 5.97%, respectively; *P*=.11), but much lower than ED (43.06%; *P*<.001), and somewhat lower than UCC (8.77%; *P*<.001) and PCP groups (11.26%; *P*<.001). While lab and imaging rates differed by condition, the pattern of lower rates of lab and imaging testing in virtual episodes (except similar rates to RHC imaging) was consistent across conditions (see Table 3).

Overall antibiotic fills within 3 days for the 6 most commonly treated infections (excluding influenza) was somewhat higher in the virtual group (70.51%) compared with all other sites (RHC 64.18%; *P*<.001, UCC 67.94%; *P*=.02, ED 56.73%; *P*<.001, PCP 68.19%; *P*=.03; Table 3), although there was variation by infection type. Fill rates after virtual visits tended to follow more similar patterns to UCC (for conjunctivitis, URI, and UTI) and PCP (for conjunctivitis, sinusitis, and URI) than RHC (similarity to sinusitis only) and ED (similarity to bronchitis only). Antibiotic fills were substantially higher after virtual visits than all other locations for pharyngitis (virtual 74.3%, RHC 29.6%; *P*<.001, UCC 53.8%; *P*<.001, ED 46.4%; *P*<.001, PCP 53.7%; *P*<.001).

Broad-spectrum antibiotics were used as first-line treatment in the virtual group (68.99%) at a similar rate to the PCP group (69.28%; *P*=.82), but more often than in the RHC (59.98%; *P*<.001), UCC (66.28%; *P*=.04), and ED groups (61.90%; *P*<.001).

### Cost Comparisons

Total costs per episode were $36, $153, $1735, and $162 more expensive at RHC, UCC, ED, and PCP settings, respectively, compared with virtual visits (Tables 4 and 5).

**Table 4:** Cost of retail health clinic and urgent care center visits compared with virtual visits, adjusted for age categories and baseline comorbidities.

	Virtual		RHC			UCC		
	n	Mean, $	n	Mean, $ (95% CI)^a^	Relative (95% CI)^b^	n	Mean, $ (95% CI)^a^	Relative (95% CI)^b^
Index visit	4635	49	13,832	74 (72-75)	1.52 (1.49-1.54)	13,757	134 (131-136)	2.75 (2.70-2.79)
Follow-up, medical	4635	200	13,832	204 (189-220)	1.02 (0.95-1.10)	13,757	266 (247-287)	1.33 (1.23-1.43)
Pharmacy	3182	90	7518	97 (91-104)	1.08 (1.01-1.15)	7188	92 (86-98)	1.03 (0.96-1.09)
Total (sum, estimate)		339		375	1.11		492	1.45

^a^Mean cost, adjusted to virtual visit distribution of age and comorbidities.

^b^Relative = ratio of how much more expensive RHC/UCC visits are compared with virtual visits after adjustments.

**Table 5:** Cost of emergency department and primary care physician visits compared with virtual visits, adjusted for age categories and baseline comorbidities.

	Virtual		ED			PCP		
	n	Mean, $	n	Mean, $ (95% CI)^a^	Relative (95% CI)^b^	n	Mean,$ (95% CI)^a^	Relative (95% CI)^b^
Index visit	4635	49	13,840	1404 (1381-1428)	28.87 (28.39-29.36)	13,881	109 (107-111)	2.25 (2.21-2.28)
Follow-up, medical	4635	200	13,840	584 (542-631)	2.92 (2.70-3.15)	13,881	288 (267-311)	1.44 (1.33-1.55)
Pharmacy	3182	90	7227	86 (81-92)	0.96 (0.90-1.02)	7629	104 (97-110)	1.15 (1.08-1.23)
Total (sum, estimate)		339		2074	6.12		501	1.48

^a^Mean cost, adjusted to virtual visit distribution of age and comorbidities.

^b^Relative = ratio of how much more expensive ED/PCP visits are compared with virtual visits after adjustments.

**Figure 2:**
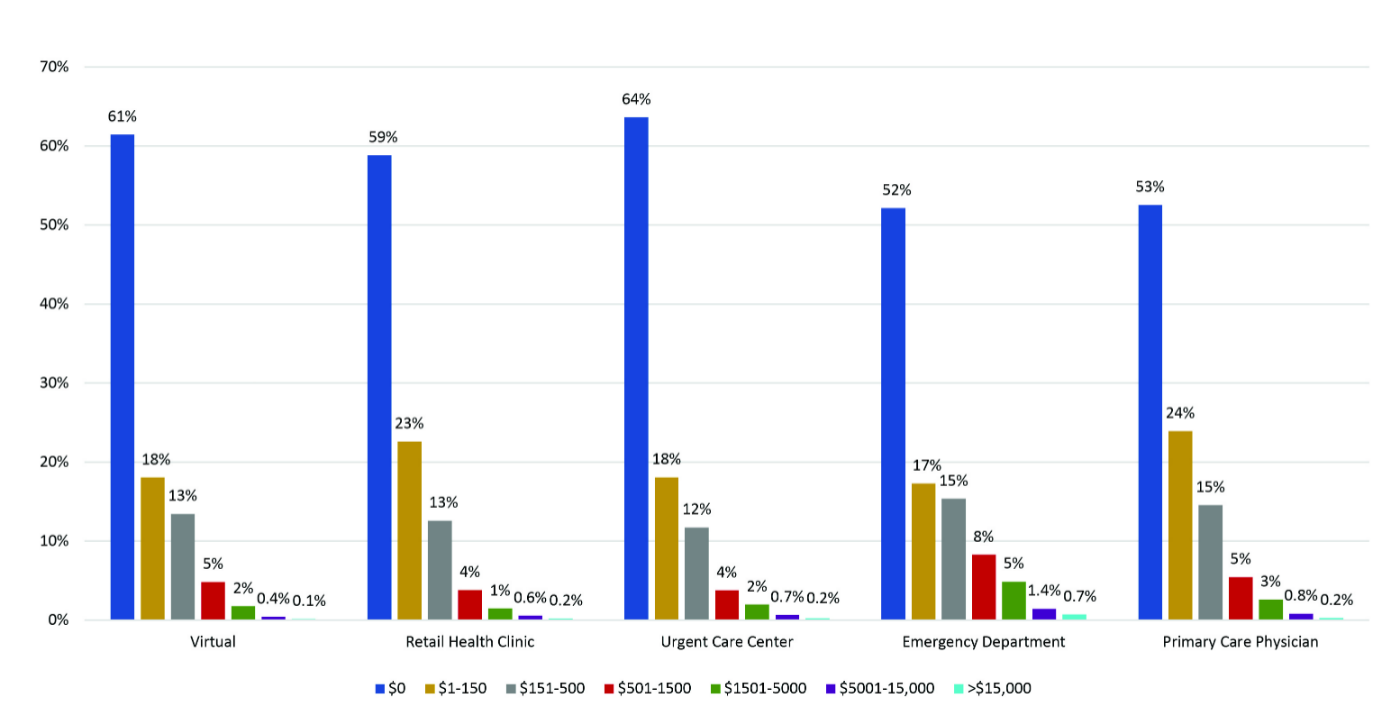
Follow-up medical costs, unadjusted.

As expected, the adjusted mean cost of the initial visit was lower for the virtual group ($49) than for RHC ($74; *P*<.001), UCC ($134; *P*<.001), ED ($1404; *P*<.001) and PCP ($109; *P*<.001; Tables 4 and 5). Follow-up medical costs for the virtual group were similar to or lower than the costs for each of the other sites of care. In the virtual group, 61.47% of patients had no follow-up medical costs, compared with 58.82% RHC, 63.63% UCC, 52.14% ED, and 52.53% PCP (Figure 2). Follow-up costs exceeded $500 for 7.08% of patients in the virtual group, compared with 6.01% RHC, 6.59% UCC, 15.23% ED, and 9.04% PCP; follow-up costs exceeded $5000 for 0.54% in the virtual group compared with 0.74% RHC, 0.88% UCC, 2.11% ED, and 1.03% PCP. Adjusted average follow-up medical costs were similar between the virtual ($200) and RHC groups ($204; *P*=.62) but higher for the UCC ($266; *P*<.001), ED ($584; *P*<.001), and PCP groups ($288; *P*<.001). At $90, the adjusted average pharmacy cost for a virtual episode was similar to UCC ($92; *P*=.44) and ED ($86; *P*=.21) and somewhat lower than RHC ($97; *P*=.02) and PCP ($104; *P*<.001).

While average episode costs differed by condition, they tended to follow a similar pattern of virtual visits having lower medical costs than care at other locations across conditions (Multimedia Appendix 2). Additionally, the sensitivity analysis with winsorized values showed consistent relative costs of care between virtual and the other locations as the original analysis, except for lower RHC and UCC follow-up medical costs, although total episode cost differences were consistent (Multimedia Appendix 3).

## Discussion

### Principal Findings

This retrospective, real-world analysis demonstrated that care received through virtual visits for nonurgent conditions was comparable to that received in in-person health care settings. Patients receiving care through virtual visits had similar follow-up outpatient evaluation and management visit rates as patients using other locations. This finding suggests not only that patients using virtual visits had their health problems resolved at similar rates as patients treated at other locations but also that patients were not using virtual visits as a first step before seeking in-person care. Interestingly, follow-up visit rates for the virtual group mirrored patients’ self-reported resolution of symptoms. An informal survey administered as part of the health plan’s virtual care program found 79% of patients who used it reported complete resolution of their health care concerns (personal communication, W Adamson).

Lab testing rates, both overall and at the individual diagnosis level, were lower during virtual visits episodes than all in-person settings. Lab testing may be high at in-person locations for some conditions where it may not be needed to confirm the patient’s diagnosis [17,18]. However, the large differences between lab testing rates for pharyngitis and UTI suggest that patients with virtual care visits may not receive testing for these conditions where differentiating between a viral and bacterial infection is important for treatment. Antibiotics for the infections most commonly diagnosed through virtual visits were prescribed significantly more often after virtual visits than any of the other in-person treatment settings. The difference between virtual visits and other settings was even greater for pharyngitis, presumably because streptococcal infection was not ruled out with a lab test. While the use of broad-spectrum antibiotics was similar between virtual visits and PCP, it was higher than the other in-person settings.

Episodes for patients who sought care at any of the in-person settings were more expensive than similar episodes beginning with a virtual visit. In addition to the virtual visit itself being less expensive than in-person visits, follow-up medical costs were lower after virtual visits than all other locations except for RHCs. Some of the lower episode costs can be attributed to lower rates of ED or inpatient follow-up care in addition to lower laboratory and imaging rates during the episode.

### Limitations

A unique strength of this study was the large database allowing for a 3:1 match of episode-based care received in a number of alternative settings, but the exclusive use of claims data introduced several limitations. The accuracy of the diagnosis in claims may be a particular concern for virtual visits, where it may be more difficult for providers to diagnose a condition without a physical examination or supporting laboratory tests. Such errors not only may lead to inaccuracies in cost comparisons, but may also affect care patterns. It is not possible to determine disease severity from a diagnosis code alone, so cases seen in the ED, for example, may have been more severe, requiring more treatment than an average case handled by a virtual visit. Furthermore, claims do not provide complete information on the reasons patients chose a specific site of care. While patients may have chosen the ED because they perceived it to be the most convenient option even for a minor illness, it is also possible they believed their condition was severe and required urgent medical attention. However, the conditions included in this study tend to be relatively minor and treatable in nonurgent settings. Additionally, patients who chose virtual visits may have differed from those who chose other treatment settings in terms of their perception of the urgency of their condition, their health literacy, or their level of comfort using computers [7,8]. The analysis did not take into account whether a patient had multiple diagnoses at a single visit, which may have led to a more costly visit or additional follow-up care. Follow-up visits and costs may or may not have been related to the original visit, and we were unable to determine conclusively if a complaint was resolved at the original visit, if the follow-up visit was part of appropriate care, or if the follow-up visit represented inappropriate care-seeking behavior on the part of the patient. Additionally, claims do not provide sufficient information to determine if virtual visits were used in situations when individuals would have otherwise waited to see if the problem resolved on its own. A recent study suggested that RHCs increased utilization for low-acuity visits due to their lower price or convenience [19]. However, whether this is good or bad is a matter of perspective, since while costs may increase, the alternative care options may allow patients who may not have otherwise received appropriate care to receive the care they needed. Hence, our study focused on the difference in cost between care options, rather than potential savings of introducing virtual visits into the market or the necessity of the visits.

### Comparison With Prior Studies

The rate of antibiotic prescriptions for the conditions included here may warrant additional study. Based on current guidelines and Choosing Wisely recommendations [20-22], prescription rates may be higher than desired in a variety of care settings. The virtual visits were associated with somewhat higher rates of antibiotic prescriptions than other sites of care overall, including for conditions for which clinical guidelines typically do not recommend antibiotics as a first line of treatment [20-22], although results were mixed when considered by condition. In some cases, the higher antibiotic prescription rate may have been due to a lower rate of laboratory testing associated with virtual visits, since a bacterial versus viral diagnosis could not be confirmed by lab test during virtual visits (eg, pharyngitis, where lab test rates were particularly low for virtual visits compared to other locations). This finding is consistent with previous research, which found higher antibiotic prescribing rates for telehealth than for office visits, especially for pharyngitis, bronchitis, and UTIs [23-25]. Future telehealth programs, particularly those integrated with a medical home or used for patients with an already existing physician-patient relationship, may be able to develop workflows that incorporate lab testing and may help with antibiotic prescribing decisions.

### Conclusions

Virtual visits are growing rapidly, and our results indicate they are inexpensive alternatives to acute care administered at other locations. Patients receiving care through virtual visits seemed to have adequate clinical resolution compared with patients receiving care elsewhere, based on follow-up visit rates. Patients did receive additional care, such as laboratory testing or imaging, presumably when needed. Virtual visits did not appear to add to the total amount of care received as part of a care episode, as patients did not often seek care through telehealth plus another site for the same condition.

Expansion of virtual health care services is inevitable given the growing use of mobile devices, patient demand for immediate and convenient access to care, and the continuously growing demands on physicians’ time. The focus of further research on virtual health care should be about optimizing patient outcomes for conditions best suited for virtual visits and examining how virtual visits can be used by physicians who have an existing personal relationship with the patient.

## Appendix

Multimedia Appendix 1 International Classification of Diseases, Ninth Revision, diagnosis codes.

Multimedia Appendix 2 Adjusted mean index visit medical, follow-up medical, and pharmacy costs, by condition.

Multimedia Appendix 3 Comparing original and winsorized adjusted costs (sensitivity analysis).

## Abbreviations

CPT: Current Procedural Terminology
DCI: Deyo-Charlson Comorbidity Index
ED: emergency department
HIRD: HealthCore Integrated Research Database
ICD-9: International Classification of Diseases, Ninth Revision
NPI: National Provider Identifier
PCP: primary care physician
RHC: retail health clinic
UCC: urgent care center
URI: upper respiratory infection
UTI: urinary tract infection
